# New onset headache during delivery and postpartum: Clinical characteristics of a case series

**DOI:** 10.3389/fneur.2022.1065939

**Published:** 2022-11-29

**Authors:** Gianni Allais, Giulia Chiarle, Silvia Sinigaglia, Elena M. Mollo, Giada Perin, Francesca Pizzino, Chiara Benedetto

**Affiliations:** ^1^Department of Surgical Sciences, Women's Headache Center, University of Turin, Turin, Italy; ^2^Midwifery Degree Course, University of Turin, Turin, Italy

**Keywords:** delivery, headache, labor, migraine, tension-type headache, pregnancy, postpartum, women

## Abstract

**Introduction:**

There are abundant studies on headache and migraine in women but few or none about their occurrence during labor, delivery, and postpartum (2 hours after delivery) owing to the low incidence. A headache attack can be debilitating when a woman is trying to manage labor pain. Research at our Women's Headache Center within the Department of Gynecology and Obstetrics has begun to shed light on this potential association.

**Methods:**

For the present study 474 women with singleton pregnancy were enrolled. A headache questionnaire was administered at two time points. Headache history was investigated on admission to prenatal care at 36 weeks gestation. The women were followed by a midwife who monitored labor progression and recorded the onset and features of headache pain. During examination before hospital discharge at 3 days post-delivery, the headache questionnaire was reviewed by a headache specialist who differentiated headache type according to International Classification of Headache Disorders (3rd edition) criteria.

**Results:**

Data analysis showed that 145/474 women had a history of headache: 65/145 (44.82%) reported a diagnosis of migraine. Eight reported experiencing a probable migraine attack (4 with aura) and one reported probable tension-type headache during labor or postpartum. All nine women who reported migraine/headache attack during labor had no previous history of headache or neurological illness. All had vaginal delivery. No onset of headache pain in patients with a previous history of headache was noted during delivery and postpartum.

**Discussion:**

The onset of a headache attack during labor in women who usually do not experience headache suggests other pathogenic mechanisms underlying the attack and merits further study.

## Introduction

Headache, and migraine in particular, affects more women than men (prevalence 3:1): 18% of women of childbearing age and 24% of those between age 30 and 39 years vs. 6% of age-matched men (1, 2). The stark contrast in prevalence stems mainly from the difference in hormonal patterns, especially the fluctuation in estrogen levels and their effect on neuronal sensitivity (3–6). For many women, pregnancy brings relief from usual migraine due to the higher pain threshold produced by elevated, stable levels of estrogen, progesterone, and endogenous opioids. Hormonal fluctuations characteristic of menstrual cycle disappear during pregnancy, resulting in fewer migraine triggers and greater neuronal stability (7, 8).

Between 50 and 80% of women with migraine, particularly those without aura, report the absence of migraine attacks or an improvement in symptoms starting from the second trimester of pregnancy (9, 10). In others, however, migraine attacks that worsen or appear *de novo* should be evaluated to exclude secondary headache of vascular origin (stroke, cerebral thrombosis) (11) or hypertension (preeclampsia) since migraine and pregnancy share a procoagulant state (12).

There are abundant studies on headache and migraine in women but none on the onset of tension-type headache (TTH) and migraine during labor and the postpartum period (2 h after delivery) owing to the low incidence. Several headache attacks during the last week of pregnancy and labor have been attributed to preeclampsia (13), cerebrovascular events (14) or dural puncture for spinal and peridural anesthesia (15). To date, there is no published evidence for the influence of headache on labor progression or on pain reliever efficacy or mode of delivery. However headache onset during labor can be extremely debilitating when a woman is in labor pain; a headache attack can add to her suffering during labor and delivery.

To fill this gap, research at our Women's Headache Center within the Department of Gynecology and Obstetrics has begun to shed light on this potential association. This research describes the characteristics of headache onset at delivery and during postpartum. The aim of our study was to draw attention in particular to migraine episodes, which are a known risk factor for cerebrovascular events and preeclampsia (6, 11, 12).

## Materials and methods

Data were collected between June 2020 and March 2021 at the Department of Gynecology and Obstetrics, Sant'Anna Hospital, Turin. Inclusion criteria were: singleton pregnancy with the fetus in cephalic presentation; no contraindications to spontaneous delivery; mother's age 18–50 years; ability to understand Italian. On admission to prenatal care at 36 weeks gestation, patient demographics and course of pregnancy were recorded. Study participants were administered a headache questionnaire-part 1 (Table 1) investigating headache history.

**Table 1:** Headache questionnaire part 1: History.

1. Do you have or have you ever had headache attacks?
(A) Yes
(B) No. If no, go to Question 1, Part 2.
2. Has the cause of the attacks been identified (for example, trauma or sinusitis)?
(A) No
(B) Yes. If yes, please specify
3. How many headache attacks have you had in your life?
(A) 1
(B) 2–5
(C) 6–10
(D) More than 10
4. The start of attacks was triggered by one of the following life events:
(A) First menstruation
(B) A previous pregnancy
(C) Previous breastfeeding
(D) Other (for example, use of hormonal contraceptives)
5. How long does the headache last without taking a painkiller?
(A) In seconds
(B) In minutes
(C) In hours
(D) In days
6. During the present pregnancy, the headache attacks have:
(A) Improved
(B) Totally remitted
(C) Remained unchanged
(D) Worsened
7. What type of pain do you usually have?
(A) Throbbing
(B) Not throbbing
8. Do you feel pain on one side or both?
(A) One side
(B) Both
9. The pain intensity is usually:
(A) Severe
(B) Moderate
(C) Mild
10. Normal activities of daily living and mild exertion:
(A) worsen the headache attack
(B) improve or have no effect on the headache attack
11. Do you feel nauseous during a headache attack?
(A) No
(B) Yes
12. Have you ever vomited during a headache attack?
(A) No
(B) Yes
13. Does bright light hurt your eyes during a headache attack?
(A) No
(B) Yes
14. Does loud noise hurt your ears during a headache attack?
(A) No
(B) Yes
15. Which of these painkillers do you usually take for a headache attack?
(A) Paracetamol
(B) Triptans
(C) Non-steroidal anti-inflammatory drugs
16. Do you experience visual disturbances during or just before the start of a headache (for example, colored lights, black spots, changes in field of vision)?
(A) No
(B) Yes. If yes, describe the disturbance.
17. Do you feel pins and needles or numbness during a headache attack?
No
Yes. If yes, specify where.
18. Is it difficult for you to speak before or during during a headache attack?
(A) No
(B) Yes
19. How long does each symptom last?
(A) In seconds
(B) In minutes
(C) In hours
(D) In days

The women were followed by a midwife who monitored labor progression and recorded the onset and features of headache pain. The midwives were instructed in administration of the headache questionnaire-part 2 (Table 2) for documenting the features of headache attacks during labor and describe the event correctly. During the hospital discharge examination a headache specialist reviewed the questionnaire items (parts 1 and 2) and completed the diagnosis based on International Classification of Headache Disorders (3rd edition) criteria (ICHD) (16).

**Table 2:** Headache questionnaire part 2: During labor/delivery/postpartum.

1. Did you have a headache attack during labor/delivery/postpartum?
(A) Yes
(B) No
2. When did the headache pain start?
(A) during the first stage of labor
(B) during the second stage of labor
(C) during the third stage of labor
(D) during postpartum
3. What type of pain did you have?
(A) Throbbing
(B) Not throbbing
4. Did you feel pain on one side or both?
(A) One side
(B) Both
5. The pain intensity was:
(A) Severe
(B) Moderate
(C) Mild
6. How long did the headache attack last?
(A) In seconds
(B) In minutes
(C) In hours
(D) In days
7. Did you take a painkiller during the headache attack?
(A) No
(B) Yes. If yes, specify ….
8. Which of the following symptoms did you experience during the headache attack:
(A) Nausea
(B) Vomiting
(C) Photophobia
(D) Phonophobia

The study sample was 474 women who gave vaginal delivery either spontaneous or pharmacologically induced with prostaglandins or oxytocin or both. Women undergoing cesarean section or emergency delivery were excluded from the study to eliminate the potential cause of headache due to inadvertent perforation of the dura mater during spinal analgesia prior to surgery.

Having a midwife present during labor in the delivery room (one-to-one care) was essential to establish the exact time point at which a headache attack began: labor stage 1: dilation of the uterine cervix from 3 cm to complete dilation; stage 2: pushing and birth; stage 3: delivery of the placenta; postpartum: the first 2 h after delivery. The study was approved by the Local Ethical Committee (number 0050254) of our institution and informed, written consent was obtained from all participants.

## Results

The study sample comprised women aged between 18 and 46 years (mean 31), 141 (29.47%) of which were multiparous. The mean gestational age at delivery was 39 weeks + 3 days. Analysis of the questionnaire responses showed that nearly one-third (145/474, 30.59%) had a history of headache: 65/145 (44.82%) reported a diagnosis of migraine (87.69% without aura, 12.31% with aura) for which the majority (81.94%) took a pain reliever [paracetamol, non-steroid anti-inflammatory drugs (NSAIDs), triptans]. Migraine began at menarche in over half (57.93%). During pregnancy, 77.93% experienced improvement or total relief of attack symptoms, 8.28% reported no change, and 13.79% reported worsening of symptoms (Table 3).

**Table 3:** Patient characteristics and headache features before pregnancy.

**Characteristic**	**All (*N* = 474)**	**Headache during delivery (*N* = 9)**	**Control (*N* = 465)**
Age -years, mean	31.00	30.78	31.01
Gestational age – weeks + days	39 + 3	39 + 4	39 + 2
Parity ≥1 - no. (%)	141 (29.74)	4 (44.44)	137 (29.46)
History of headache – no. (%)	145 (30.59)	0	145 (30.59)
Previous diagnosis of migraine – no. (%)	65 (43.66)	0	65 (43.66)
Migraine without aura – no. (%)	57 (87.69)		57 (87.69)
Migraine with aura – no. (%)	8 (12.31)		8 (12.31)
Therapy	118 (81.94)	n.a.	118 (81.94)
Paracetamol – no. (%)	31 (34.07)		31 (34.07)
NSAIDs – no. (%)	36 (39.56)		36 (39.56)
Triptans – no. (%)	24 (26.37)		24 (26.37)
Onset		n.a.	
Menarche – no. (%)	84 (57.93)		84 (57.93)
Pregnancy- no. (%)	0		0
Breastfeeding – no. (%)	0		0
Others – no. (%)	61 (42.07)		61 (42.07)
Headache in pregnancy		n.a	
Improved – no. (%)	55 (37.93)		55 (37.93)
Unchanged - no. (%)	12 (8.28)		12 (8.28)
Disappeared – no. (%)	58 (40.00)		58 (40.00)
Worsened – no. (%)	20 (13.79)		20 (13.79)

n.a, not applicable; NSAIDs, non-steroidal anti-inflammatory drugs.

Analysis of responses to part 2 of the headache questionnaire revealed that nine women (1.89%), with no history of migraine or headache experienced a headache or migraine attack during labor and postpartum (Table 4). The mean age was 30.78 years, the gestational age was 39 weeks + 4 days, and four of the nine women were multiparous. Eight pregnancies were normal; gestational diabetes developed in one, which was managed with a hypoglycemic diet. Labor was spontaneous in seven women and pharmacologically induced in two (one because of post-term pregnancy and one because of gestational diabetes with fetal biometry >95th centile). All nine women gave vaginal delivery without the need for instrumental delivery or emergency cesarian section. Headache onset occurred during the labor stage 1 in seven women, during labor prodrome in one, and during labor stage 3 in another.

**Table 4:** Patient characteristics and headache features during labor, delivery, and postpartum.

	**CASE 1**	**CASE 2**	**CASE 3**	**CASE 4**	**CASE 5**	**CASE 6**	**CASE 7**	**CASE 8**	**CASE 9**
Age (years)	31	29	33	27	32	29	35	36	25
Parity	Multiparous	Nulliparous	Multiparous	Multiparous	Nulliparous	Nulliparous	Nulliparous	Multiparous	Nulliparous
History of headache	No	No	No	No	No	No	No	No	No
Gestational age (weeks + days)	40 + 2	39 + 6	41 + 4	40 + 1	39 + 1	39 + 5	38 + 5	39 + 4	40 + 3
Complications of pregnancy	Hypothyroidism	None	None	None	None	Gestational Diabetes Mellitus	None	None	None
Labor and delivery	Spontaneous, vaginal	Spontaneous, vaginal	Induction with prostaglandin, vaginal	Spontaneous, vaginal	Spontaneous, vaginal	Induction with prostaglandin, vaginal	Spontaneous, vaginal	Spontaneous, vaginal	Spontaneous, vaginal
Headache onset	Labor stage 1	Labor stage 1	Labor prodromes	Labor stage 1	Labor stage 1	Labor stage 1	Labor stage 3	Labor stage 1	Labor stage 1
Headache pain	Throbbing unilateral	Throbbing unilateral	Throbbing, bilateral	Throbbing unilateral	Throbbing unilateral	Throbbing bilateral	Non pulsating bilateral	Throbbing unilateral	Throbbing bilateral
Pain intensity	Moderate	Severe-moderate	Moderate	Severe	Severe-moderate	Moderate-mild	Mild	Severe	Mild
Associated symptoms	Nausea, photophobia, phonophobia	Nausea, vomiting photophobia, phonophobia	Nausea, photophobia, phonophobia	Vomiting, photophobia, phonophobia	Nausea, photophobia, phonophobia	Nausea	Photophobia	Nausea, vomiting, photophobia, phonophobia	Nausea, photophobia, phonophobia
Aura	No	No	Sensory (right hand) lasting 30 min	Sensory (left hand and lips) lasting 30 min	No	Visual lasting 10 min	No	Sensory (right side) lasting 30 min	No
Duration (h)	6 h	4 h	7 h	4 h	4 h	5 h	2 h	4.5 h	6 h
Resolution	Spontaneous	With paracetamol	Spontaneous	With paracetamol	Spontaneous	With Ibuprofen	With paracetamol	With paracetamol	Spontaneous
Headache type	Probable migraine without aura	Probable migraine without aura	Probable migraine with aura	Probable migraine with aura	Probable migraine without aura	Probable migraine with aura	Probable TTH	Probable migraine with aura	Probable migraine without aura

TTH, tension-type headache.

Eight women experienced a probable migraine attack according to ICHD diagnostic criteria (16): four reported aura before the attack (paresthetic episodes affecting the hand and the lips lasted 30 min in three women and visual phenomena lasted 10 min in one woman). One woman reported probable TTH, with mild constricting pain and nausea during labor stage 3. Headache attacks resolved either spontaneously or taking a painkiller (paracetamol and ibuprofen) within 24 h after onset. Clinical and laboratory assessment was performed at headache onset to rule out secondary headache due to eclampsia or acute cerebrovascular events. Subsequent clinical evaluation revealed no neurological signs or headache in any of these patients. Since none of the nine patients received epidural anesthesia, post-dural puncture headache can be ruled out in this patients. Furthermore, since none of the women had a history of headache, the single episode of headache during labor may be defined as a probable migraine or TTH. The insufficient number of headache episodes (only one) fails to meet the ICHD (16) criteria for diagnosing migraine or TTH.

## Discussion

In our syudy a probable migraine attack occurred during labor in 8/474 women (four of which with aura) and probable TTH in one. All deliveries were vaginal. The onset of headache occurred most often during labor stage 1, when the uterine cervix was dilating (probable migraine attack in seven women). None of the nine women had a history of migraine or headache. To our best knowledge, there are no published data like ours so far.

We focus our attention on migraine because of the higher prevalence of in these patients (8/9 attacks) and its important as a cardiovascular and obstetric risk factor (6, 11, 12).

Migraine attacks can be triggered by a drop in estrogen levels, as occurs during the perimenstrual phase, if preceded by exposure to elevated hormone levels, as occurs during the medial-luteal phase (17). A similar condition can be seen during puerperium (lasting 40 days after birth) when estrogen level begins to fall, with a decrease of 95% within 12 h after delivery (18). It is then that a rise in migraine incidence can be expected: during the first 24 h after birth, an increase in migraine and headache attacks has been reported by patients with a history of migraine (19). While women with a history of migraine may report headache during the first h after delivery, we were surprised to find that none in this patient subset experienced a migraine attack during labor. Our observation of headache attacks during labor in women without a history of migraine suggests that other mechanisms underlie its occurrence.

The onset of labor is accompanied by an increase in estrogen level and a shift in the estrogen/progesterone balance toward estrogen (20), setting the condition for myometrial changes that promote muscle contraction: increase in oxytocin and prostaglandin receptors, overexpression of enzymes regulating muscle contraction reactions, and a drop in calcitonin gene-related peptide levels (21, 22). Such a contractile-conducive milieu could explain the relatively high prevalence of migraine with aura in this study. In certain situations and in predisposed women, muscle contraction may involve the muscle tunica of cerebral vessels, leading to a transient drop in blood flow and migraine aura.

This study has several limitations. Long-term follow-up might have disclosed further migraine attacks or features of secondary headache in these patients. Nonetheless, this is the first study to report probable migraine and TTH attacks in women without a history of headache or associated systemic or neurological illness and no attacks in patients with a known history of headache. Assessment of symptoms by trained midwife staff caring for the patients in the delivery room strengthens the reliability of the study data. The midwives were supported by the headache specialists at our institution. Future studies comparing women without a history of migraine and those with a known history of migraine may elucidate the mechanisms underlying this finding and its real prevalence during delivery and postpartum.

This preliminary study sheds new light on women with migraine. Investigation into the still poorly understood mechanisms underlying the onset of migraine attack during labor and delivery may provide a new focus of research. Further study involving larger population and control groups is needed.

Finally, professional education of health care workers in gynecology and obstetrics about headache in women is highly desirable, given the prevalence of headache and the potential occurrence of attacks during labor and delivery. Prompt recognition of headache symptoms could minimize overreaction and overtreatment and provide the support a woman really needs in such circumstances.

## Data availability statement

The raw data supporting the conclusions of this article will be made available by the authors, without undue reservation.

## Ethics statement

The studies involving human participants were reviewed and approved by Ethics Committee Città della Salute e della Scienza, Torino. The patients/participants provided their written informed consent to participate in this study.

## Author contributions

GA conceived the study, supervised data records, reviewed headache questionnaire, and data and reviewed manuscript. GC reviewed headache data and their analysis, and wrote the manuscript. SS reviewed headache data and their analysis. EM supervised midwives. GP and FP followed patients during labor and collected headache data. CB supervised manuscript production. All authors contributed to the article and approved the submitted version.

## Conflict of interest

The authors declare that the research was conducted in the absence of any commercial or financial relationships that could be construed as a potential conflict of interest.

## Publisher's note

All claims expressed in this article are solely those of the authors and do not necessarily represent those of their affiliated organizations, or those of the publisher, the editors and the reviewers. Any product that may be evaluated in this article, or claim that may be made by its manufacturer, is not guaranteed or endorsed by the publisher.
